# Growth Performance, Biochemical Blood Indices, and Large Intestine Physiology of Rats Fed Diets with Alfalfa Protein-Xanthophyll Concentrate

**DOI:** 10.3390/ani11072069

**Published:** 2021-07-12

**Authors:** Marcin Barszcz, Anna Tuśnio, Ilona Bachanek-Matusiewicz, Kamil Gawin, Jacek Skomiał, Marcin Taciak

**Affiliations:** Department of Animal Nutrition, The Kielanowski Institute of Animal Physiology and Nutrition, Polish Academy of Sciences, 05-110 Jabłonna, Poland; a.tusnio@ifzz.pl (A.T.); i.bachanek@ifzz.pl (I.B.-M.); k.gawin@ifzz.pl (K.G.); j.skomial@ifzz.pl (J.S.); m.taciak@ifzz.pl (M.T.)

**Keywords:** alfalfa, xanthophylls, blood indices, fermentation, liver, colon, mucus

## Abstract

**Simple Summary:**

Alfalfa protein-xantophyll concentrate is a rich source of nutrients and bioactive compounds. It provides protein, lutein, vitamins, minerals, phytoestrogens, and other plant metabolites. Thus, it may affect animal health in many ways; however, its impact is not fully recognized. Therefore, the aim of the study was to determine the effect of dietary supplementation with 1.5% and 3% concentrate on growth performance, blood biochemical profile, and large intestine physiology of rats as a model animals. The results showed that feeding a diet supplemented with alfalfa concentrate could reduce thickness of the protective mucus layer in the colon of rats but did not affect animal growth and microbial activity in the caecum.

**Abstract:**

The effect of dietary levels of alfalfa protein-xanthophyll concentrate (PXC) was determined in growing rats. Three groups of eight four-week-old male Wistar rats, with an average initial body weight of 61 g, were fed for 28 days either natural-ingredient diets without PXC or supplemented with 1.5% or 3% PXC. Growth performance, blood biochemistry, caecal fermentation, morphology of the large intestine, and mucin gene expression were evaluated. PXC did not affect growth performance but tended to decrease relative liver weight. Among biochemical blood parameters, only bilirubin decreased and uric acid increased in response to 1.5% and 3% PXC, respectively. Caecal fermentation was not affected, with the exception of isovaleric acid concentration, which tended to be higher in rats fed the diet containing 3% PXC. Colonic crypts tended to be deeper in rats fed the 3% PXC diet and the thickness of the colonic mucus layer was reduced by both PXC levels. In conclusion, PXC did not affect growth performance or caecal fermentation but decreased thickness of the protective mucus layer in the colon.

## 1. Introduction

Alfalfa (*Medicago sativa* L.) is a valuable legume, whose protein yield (2–3 tonnes per hectare) is several times greater than that of other legumes, such as soybean and pea, but also grains [1]. However, alfalfa cannot constitute the major protein source in feeds intended for young monogastric animals due to its high crude fibre content, reaching 23–30% dry matter [1,2]. Other factors limiting the use of alfalfa in animal nutrition include low content of digestible energy, antinutritional factors, low digestibility of protein in meal, and decreased palatability at high levels in feed [3]. A high reduction of crude fibre content (to 1–2%) can be achieved by separating protein from fibre during the production of protein-xanthophyll concentrate (PXC). In this process, alfalfa is pulped and pressed to obtain a green juice, which is subsequently heated at 85–90 °C to coagulate proteins. Subsequently, the coagulum is separated from the residual solution, dried, supplemented with antioxidant, and pelleted. The obtained PXC, in addition to the reduced fibre content, contains about 55% protein and over 1200 mg/kg of xanthophylls (lutein, zeaxanthin, violaxanthin, and neoxanthin) [1]. The protein of concentrates produced from alfalfa is highly digestible [4] and readily accepted by rats [3,5]. Alfalfa protein is well utilized by growing-finishing pigs and it provides results comparable to soybean meal, even when used as the only protein supplement in a barley-based diet [5].

The PXC is a rich source of minerals, particularly calcium (32 mg/g), as well as easily available iron (0.7 mg/g) and vitamins such as β-carotene (92 µg/g), tocopherol (0.3 mg/g), vitamin K (30 µg/g), and folic acid (3 µg/g) [1]. The concentrate also contains many secondary metabolites and the most important are: saponins, phytoestrogens (isoflavones and coumestrol), phytates, and L-canavanine [2]. Owing to its rich chemical composition, PXC may affect animal physiology in numerous ways. It was shown that feeding diets supplemented with 1.5% and 3% PXC decreased the activity of superoxide dismutase and catalase, and affected the total antioxidant status in the lamb serum depending on the dietary level and animal’s age [6]. This supplement was demonstrated to positively affect haematological parameters of lambs, nutrient utilization by cattle, meat deposition in growing pigs and pork fatty acid profile, as well as to decrease cholesterol level in pig blood [2]. It was also found that PXC contributed to higher body weight of sows in late gestation and birth weight of piglets, as well as to a higher number of liveborn and weaned piglets [7]. Supplementation of broiler chicken diets with 1.5% and 3% PXC increased crude protein content in breast and thigh muscles, and the 3% PXC diet reduced cholesterol and triacylglycerol concentration, and improved antioxidant parameters in blood [8]. As a feed additive for laying hens, PXC increased the intensity of egg yolk colour, which could attract consumer interest, leading to higher egg consumption [9]. The addition of PXC, particularly at the dose of 3%, may also be used to increase the total concentration of polyunsaturated fatty acid in egg yolks both from n-3 (linolenic and docosahexaenoic acid) and n-6 (linoleic acid) family, and increase the n-3 to n-6 ratio [10]. The health-promoting potential of PXC may also be utilized in combating iron and folic acid deficiencies in anaemia patients [11]. However, the technique of PXC production should be modified to improve its functional properties for humans and increase consumer acceptance because the dark colour and bitter taste limit its application [12].

In the available literature, there is no information about the impact of PXC on the physiology of the gastrointestinal tract. Microbial activity in the large intestine is of particular importance, as intestinal bacteria are involved in the regulation of many metabolic pathways initiating host–microbe interactions connecting the intestine, liver, muscles, and brain [13]. Modification of diet composition results in changes in the influx of substrates for the microflora of the large intestine [14]. Thus, feeding a diet supplemented with PXC, which contains a wide range of bioactive compounds, may influence microbial activity in the large intestine and, consequently, animal physiology. Therefore, the aim of the study was to evaluate the effect of dietary supplementation with PXC on the growth performance, blood biochemical profile, and large intestine physiology of rats as model animals.

## 2. Materials and Methods

Study design, animal care, and experimental procedures were approved by the Third Local Animal Experimentation Ethics Committee (Warsaw University of Life Sciences-SGGW, Warsaw, Poland) under permit number 64/2011, in accordance with the principles of the European Union and the Polish Animal Protection Act.

### 2.1. Animals and Diets

The experiment was performed on 24 four-week-old male Crl:W (Han) Wistar rats (Medical University of Bialystok, Bialystok, Poland) divided into 3 groups (n = 8) so that the mean initial body weight in each group was similar (61 g). The animals were fed a commercial, cereal-based diet for laboratory animals (Labofeed H, Morawski Feed Production Plant, Żurawia, Poland) without PXC or supplemented with 1.5% or 3% PXC (Luzerne-Recherche et Développement, Chalons en Champagne, France), which was added to the diet instead of soybean meal (Table 1). Rats were kept individually in wire-bottom metabolic cages, under controlled conditions of 22 ± 1 °C and a 12 h dark-light cycle, with free access to feed and water. Body weight and feed intake were measured weekly. After 28 days of feeding, all rats were anaesthetised by isoflurane inhalation, blood samples were collected into heparinised tubes by intracardiac puncture, and the plasma was stored at −40 °C for further analyses. Internal organs (heart, spleen, liver, pancreas, stomach, small intestine, caecum, and kidneys) were excised and weighed. Caecal digesta of each rat was also weighed and samples were taken for analyses of pH, short-chain fatty acids (SCFA), amines, and bacterial β-glucuronidase. Digesta samples for the SCFA assay were stored at −20 °C, and for amine and β-glucuronidase analyses at −80 °C. Tissue samples for histological examination were taken from the caecum and colon, rinsed with 0.9% NaCl and placed in Bouin’s solution. The middle part of the colon was removed and placed in Krebs–Henseleit buffer to determine the thickness of the mucus adherent layer. Colon samples for gene expression analysis were rinsed with 0.9% NaCl, blotted dry, snap-frozen in liquid nitrogen, and stored at −80 °C.

### 2.2. Nutrient Analyses

The nutrient contents of the experimental diets were determined according to the standard procedures of the Association of Official Analytical Chemists [15]; gross energy of the diets was measured using a Parr adiabatic oxygen bomb calorimeter (KL-10, Precyzja, Poland).

### 2.3. Blood Biochemistry

Blood plasma biochemical parameters were determined spectrophotometrically using a MAXMAT PL multidisciplinary diagnostic platform (Erba Diagnostics France SARL, Montpellier, France) and ELITech ready-to-use reagents (ELITech Group, Puteaux, France).

### 2.4. Measurement of Digesta pH and SCFA Analysis

Caecal digesta pH was measured using a WTW pH/340 pH-meter (WTW GmbH, Weilheim, Germany). For SCFA analysis, the digesta (about 4.0 g) was mixed with an ultra-pure water, alkalised to pH 8.2 using 1 M NaOH, and centrifuged (1000 rpm, 10 min, room temperature). The supernatants were stored at −20 °C. SCFA concentrations were determined using a HP 5890 Series II gas chromatograph (Hewlett-Packard, Waldbronn, Germany) with a flame-ionization detector, Supelco Nukol fused silica capillary column (30 m × 0.25 mm i.d.; 0.25 µm) and isocaproic acid as an internal standard [16].

### 2.5. β-Glucuronidase Activity Assay

Caecal digesta samples (0.5 g) were placed in ice-cold potassium phosphate buffer (pH 6.8 at 37 °C), homogenised for 30 s at high speed, sonicated (2 × 30 s), and centrifuged (10,000 rpm, 20 min, room temperature). The supernatants were stored at −80 °C until analysis. β-glucuronidase activity was analysed spectrophotometrically according to the method described previously using phenolphthalein β-D-glucuronide as a substrate [16]. Absorbance was measured at 540 nm using a Unicam UV 300 spectrophotometer (Thermo-Spectronic, Cambridge, UK) and the activity of β-glucuronidase was calculated based on a standard curve prepared for phenolphthalein.

### 2.6. HPLC Analysis of Amines

The concentration of amines in caecal digesta was determined using the HPLC method described previously [17]. The digesta samples were homogenised in ultra-pure water by intensive vortexing and supernatants were obtained by centrifugation at 10,000 rpm for 15 min. The supernatant was subsequently diluted five-fold with an acetone and water mixture (2:1) and alkalised with borax buffer (0.1 M, pH 10.5). Heptylamine was then added as an internal standard to a final concentration of 5 μg/mL. Subsequently, amines were derivatised by incubation with 1% dansyl chloride in acetone for 25 min at 65 °C in a water bath in the dark and then extracted using Waters SEP-PAK serif™ C18 cartridges for solid phase extraction (6 mL, 500 mg; Waters, Watford, Hertfordshire, UK). Separation was carried out using a Finnigan Surveyor Plus HPLC (Thermo Scientific, San Jose, CA, USA) with a photodiode array detector and a Waters Symmetry Shield RP18 column (150 × 3.9 mm i.d., 5 μm) preceded by a guard column (Waters Symmetry Shield RP18, 20 × 3.9 mm, 5 μm). The mobile phase was composed of 5% acetonitrile in water and 100% acetonitrile, flowing under a gradient elution. Dansyl derivatives of amines were detected by measuring absorbance at 254 nm, and their concentrations were determined from standard curves.

### 2.7. Large Intestine Histology

Paraffin-embedded caecal and colonic tissue samples were cut into 5-µm sections using a rotary microtome. Subsequently, the slides were deparaffinised with xylene, hydrated, and finally stained with haematoxylin and eosin. Crypt depth and myenteron thickness (15 measurements per slide, 2 slides per sample) were determined using a Zeiss Axio Star Plus light microscope (Carl Zeiss, Göttingen, Germany) and Axio Vision LE Rel. 4.5 image analysis software (Carl Zeiss, 2002–2005).

### 2.8. Measurement of Mucus Layer Thickness

The middle colon segment was cut into 0.5 × 1 cm portions and the thickness of the mucus adherent layer was estimated spectrophotometrically based on the quantity of alcian blue dye absorbed by colonic tissue [18]. The absorbance was measured at 620 nm using a Unicam UV 300 spectrophotometer. The quantity of the absorbed dye was calculated from a standard curve for alcian blue.

### 2.9. Total RNA Isolation

Total RNA was extracted from colon samples using the NucleoSpin^®^ RNA II kit (Macherey-Nagel GmbH & Co. KG, Düren, Germany). The integrity of the isolated RNA was checked by electrophoresis on a 2% agarose gel stained with ethidium bromide. RNA concentration and purity were analysed using a NanoDrop ND-1000 spectrophotometer (Thermo-Scientific, Wilmington, DE, USA).

### 2.10. Analysis of Mucin Gene Expression

The expression of mucin genes (MUC2 and MUC3) and β-actin gene were examined by real-time quantitative PCR. The TRANSCRIPTME RNA kit (BLIRT SA DNA-Gdansk Division, Gdańsk, Poland) was used for reverse transcription and cDNA synthesis. PCR amplification of target genes was performed using previously published [19] specific primers (Table 2) and the QUANTUM EvaGreen^®^ PCR Kit (Syngen biotech Ltd., Wrocław, Poland). The qPCR reaction was performed in a total reaction volume of 20 μL and it contained: 3 μL of cDNA (150 ng), 4 μL of 5 × Quantum EvaGreen^®^ Mix, 0.5 μL of each primer and 12 μL of nuclease-free water. Reactions were carried out using a MIC qPCR thermocycler (Bio Molecular Systems, Upper Coomera, QLD, Australia) according to the following amplification program: 15 min of enzyme activation and initial denaturation at 95 °C, and 40 cycles of denaturation (15 s, 95 °C), annealing (20 s, 60 °C), and elongation (20 s, 72 °C). Relative gene expression was calculated based on the cycle threshold value (Ct) using the 2^−ΔΔCt^ method [20] and β-actin as the reference gene. The stability of the reference gene was checked and it did not change under the experimental conditions.

### 2.11. Statistical Analyses

All data, except for the qPCR results, were analysed using one-way analysis of variance followed by Tukey’s HSD post hoc test to determine differences between the groups. Orthogonal polynomial contrasts were used to analyse the responses to different PXC levels. All analyses were performed using the Statgraphics Centurion XVI ver. 16.1.03 statistical package (StatPoint Technologies, Inc., Warrenton, VA, USA). The qPCR results were analysed using a two-tailed Student’s t-test implemented in the micPCR software 2.10 (Bio Molecular Systems, Upper Coomera, QLD, Australia). The effects were considered significant at *p* ≤ 0.05 and a tendency was set at *p* ≤ 0.10.

## 3. Results

All animals appeared to be healthy before and during the experiment and no medical pretreatment was necessary. Feeding diets supplemented with PXC did not affect feed intake, body weight gain, or feed efficiency. There was also no effect of PXC on the relative weight of the heart, pancreas, stomach, small intestine, caecum, spleen, or kidneys, but the relative weight of the liver tended to be lower in rats fed diets supplemented with PXC (*p* < 0.10) as compared to rats fed the control diet (Table 3). There was a significant linear effect of the PXC level on the relative liver weight (*p* < 0.05).

Dietary inclusion of PXC did not affect most of the analysed biochemical blood parameters, except for uric acid and total bilirubin concentrations. Rats fed the diet containing 3% PXC had significantly higher (*p* < 0.05) uric acid concentration than animals fed other diets. The analysis of orthogonal polynomial contrasts revealed that the effect of PXC level on uric acid concentration was both linear and quadratic, and the latter model was better fitted. Feeding the diet containing 1.5% PXC resulted in a lower (quadratic effect, *p* < 0.05) total bilirubin concentration in comparison with the control group (Table 4).

Neither total SCFA nor the concentrations of individual acids were affected by the PXC although there was a tendency (*p* < 0.10) towards a higher isovaleric acid concentration in rats fed the 3% PXC diet as compared to other groups. The response of isovalerate concentration to increasing PXC levels was linear (*p* < 0.05). The relative weight of caecal digesta, digesta pH, and activity of bacterial β-glucuronidase did not differ significantly between the groups (Table 5).

Methylamine and tyramine were the main biogenic amines present in the caecal digesta of rats. Other amines, i.e., putrescine, cadaverine, and spermidine were present at lower concentrations. Dietary supplementation with PXC had no significant effect on the concentrations of amines identified in the caecal digesta (Table 5).

**Figure 1:**
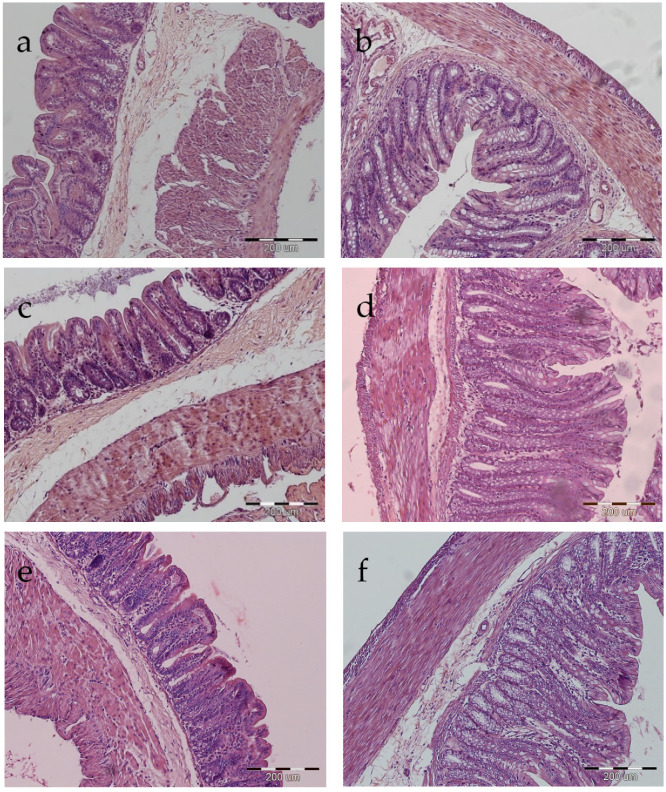
Examples of images of haematoxylin and eosin-stained section of the caecum (**a**,**c**,**e**) and colon (**b**,**d**,**f**) of rats fed the control diet (**a**,**b**) and diets supplemented with 1.5% (**c**,**d**) and 3% (**e**,**f**) alfalfa PXC.

There was no effect of PXC-containing diets on crypt depth or myenteron thickness in the large intestine. However, there was a linear effect of PXC level on crypt depth in the colon (*p* < 0.05) and a strong tendency (*p* < 0.10) towards deeper crypts in rats fed the 3% PXC diet in comparison with other groups (Table 6). Examples of images of haematoxylin-eosin-stained sections of the caecum and colon are shown in Figure 1.

The thickness of the mucus adherent layer was significantly reduced (*p* < 0.01) in the colon of rats fed PXC-supplemented diets in comparison with the control group (Table 6). The effect was both linear and quadratic (*p* < 0.01) but the latter model was better fitted to the data.

**Figure 2:**
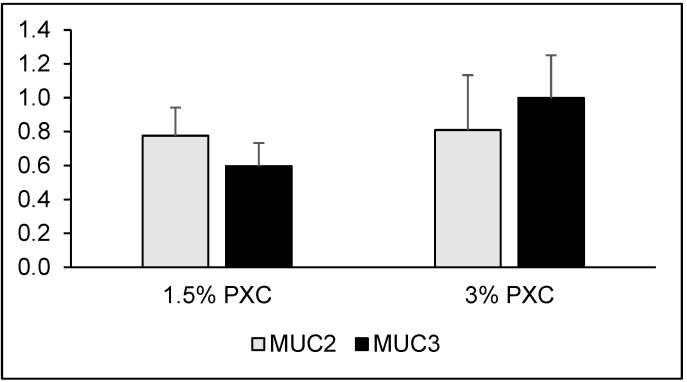
Mucin gene expression in the colon of rats fed diets supplemented with 1.5% and 3% alfalfa PXC. Significance of differences in *MUC2* gene expression: control vs. 1.5% PXC *p* = 0.174, control vs. 3% PXC *p* = 0.578. Significance of differences in *MUC3* gene expression: control vs. 1.5% PXC *p* = 0.061, control vs. 3% PXC *p* = 0.892.

Feeding diets supplemented with PXC did not affect mucin gene expression (Figure 2) although there was a tendency for downregulation of MUC3 expression associated with the 1.5% PXC diet (*p* < 0.10).

## 4. Discussion

Earlier studies on the use of alfalfa protein concentrates in diets for rats revealed that lysine and methionine were the major limiting amino acids in this protein source. Supplementation with these amino acids improves the protein efficiency ratio of the concentrate to the level of soybean or herring meal, which are characterized by high protein quality [3]. In our experiment, the diets were supplemented with crystalline amino acids and were isoproteinous and isoenergetic. They covered nutritional requirements of growing rats, therefore, no differences in growth performance were observed due to PXC supplementation.

Dietary protein content affects internal organ weights in growing animals, leading to hypertrophy at higher dietary levels [21,22]. In our study, no organ hypertrophy was observed due to an equal protein content in the experimental diets. A slightly lower relative liver weight in rats fed PXC-supplemented diets could indicate a less intensive liver metabolism and a hepato-protective role of the PXC. This positive impact of PXC has been corroborated by total blood bilirubin concentration, which was decreased by PXC at the 1.5% inclusion level. Bilirubin, a product of haem catabolism, is conjugated in the liver with glucuronic acid to increase its solubility, and then it is excreted in the bile to the small intestine [23]. Thus, a lower plasma concentration of bilirubin indicates its effective removal from the blood and confirms proper liver function. The effect of PXC on the liver could result from a high content of xanthophylls. A similar effect was described for astaxanthin, a red xanthophyll pigment of microbiological origin, which inhibited body weight gain, adipose tissue and liver weights, and decreased blood parameters related to lipid metabolism in mice fed a high-fat diet [24].

In our experiment, PXC supplementation did not affect cholesterol, triglycerides, or high-density lipoprotein concentrations in the blood because all diets were isoenergetic and fat content did not exceed the nutritional requirements of rats. Other biochemical indices were also not affected, except for uric acid concentration, which was increased by feeding the diet containing 3% PXC. Uric acid is the major degradation product of endogenous and exogenous purine bases. Purine nucleosides (adenosine and guanosine) of dietary origin belong to non-protein nitrogenous compounds, which constitute 20% of PXC protein [1]. Increased PXC levels in the diet leads to a greater supply of purines, which are subsequently metabolised to uric acid and may lead to its higher levels in the blood. However, this mechanism is rather unlikely due to the low level of dietary supplementation.

Higher uric acid concentration may contribute to the improved blood antioxidant status. Earlier studies on lambs [6] showed that PXC increased the ferric reducing activity of plasma (FRAP). Uric acid is the main compound contributing to FRAP because it can scavenge singlet oxygen and hydroxyl radicals and suppress lipid peroxidation in the erythrocyte membrane, which is probably involved in red blood cell aging [25]. The antioxidant properties of PXC result from the free radical-scavenging activity of alfalfa leaf peptides, which are chelating agents of transition metal ions [26], and the high content of xanthophylls, which are also potent antioxidants [27]. Unfortunately, in our study, blood antioxidant status was not analysed and it can only be hypothesised that antioxidant properties of PXC also result from its effect on blood uric acid level.

Feeding diets containing PXC did not affect any of the indices of microbial activity in the caecum of rats. PXC protein is highly digestible [4], therefore it cannot be utilized by the caecal microflora as a source of nitrogen and it does not intensify proteolytic fermentation leading to the production of branched-chain fatty acids (BCFA) and amines [28]. The trend towards increased concentrations of isovaleric acid, which is a product of bacterial leucine catabolism [28], may only reflect the higher content of leucine in PXC-supplemented diets, as it is the major amino acid in alfalfa leaf concentrate [1].

The lack of differences in other microbial activity indices could be also explained by crude fibre content, which was almost the same in the experimental diets and did not differ substantially due to PXC addition. Indigestible carbohydrates, present in a very small amount in PXC [1], do not exert a bulking effect on digesta and cannot serve as an energy source for the microflora. Fermentation pattern and activity of bacterial β-glucuronidase were also not affected by secondary plant metabolites present in PXC. Despite the well-documented antibacterial activity of saponins [29,30], their content in PXC does not exceed 1% [1] and is probably too low to influence microbial activity in the caecum of rats.

The diets supplemented with PXC did not alter the histological parameters of the large intestine of rats, with the exception of crypt depth in the colon. The strong tendency of PXC to increase crypt depth, especially at the 3% dietary level, could be associated with liver function. The liver produces most of insulin-like growth factor-I (IGF-I) circulating in the blood and its synthesis is stimulated by growth hormone [31]. IGF-I is involved in colonic cell proliferation, apoptosis inhibition, and is necessary for cell cycle progression. It has also been found that the proliferating zone in the colonic crypts is extended at high IGF-I concentrations, thus IGF-I may be involved in colon carcinogenesis [31]. Growth hormone level rises at low blood concentrations of liver-derived IGF-I, leading to increased liver weight through a feedback regulation [32]. In our study, we found that liver weight in rats tended to be lower due to PXC supplementation and it was assumed that PXC components were able to stimulate liver IGF-I production, which in turn led to the intensification of colonic cell proliferation and deeper crypts. Another mechanism responsible for the effect of PXC on crypt size could be associated with greater bile flow from the liver to the intestine. Bile is a carrier of bilirubin glucuronides, which are substrates for β-glucuronidase [33] but also of bile acids conjugated to glycine or taurine [34]. Bile acids are emulsifiers necessary for the absorption of xanthophylls [35], which are present in high quantities in PXC. Despite highly efficient bile acid transport in the ileum, about 5% of these compounds escape the enterohepatic circulation and are metabolised by bacteria in the large intestine. Taurine contains a sulphonic acid moiety, which is transformed to hydrogen sulphide after deconjugation. Hydrogen sulphide is a toxic agent, which increases colonocyte turnover and induces epithelial cell proliferation [34]. This may be the underlying mechanism for the deeper colon crypts, observed following PXC supplementation.

Hydrogen sulphide might also have contributed to a thinner mucus layer in the colon of rats fed the PXC-supplemented diets. The mucus layer plays the most important role in the maintenance of intestinal homeostasis and its thickness results from the balance between mucin secretion and enzymatic or mechanical degradation of these glycoproteins [18,36]. A thinner mucus layer may allow pathogenic bacteria to penetrate the mucus and attach to epithelial cells [37]. Hydrogen sulphide may damage mucin disulphide bridges, leading to impairment of the intestinal barrier [38]. It may also contribute to the reduction of the mucus layer through an inhibition of butyrate metabolism in the colonic epithelium. Butyrate stimulates mucus secretion [34,39], thus its impaired metabolism in the colon mucosa may result in a thinner mucus layer. The present study found that MUC3 expression tended to be downregulated by the 1.5% PXC diet, which was consistent with the finding that MUC3 expression was related to butyrate [40]. Unfortunately, SCFA and hydrogen sulphide concentrations were not analysed in the colon of rats and we can only speculate about the underlying mechanisms of the PXC effect. It is unknown whether the effect on the thickness of the mucus adherent layer is specific to PXC xanthophylls only, hence other dietary sources of xanthophylls should also be considered.

## 5. Conclusions

The results of the present study indicate that diets supplemented with alfalfa PXC do not influence growth performance or fermentation processes in the caecum of rats but may affect liver and colon physiology. Deeper crypts and a thinner mucus layer in the colon are probably related to higher bile secretion necessary for the absorption of xanthophylls. However, further research is required to confirm this assumption.

## Figures and Tables

**Table 1:** Composition of experimental diets for rats.

	PXC (%)		
	0	1.5	3
Components (g/kg, as-fed basis)			
Protein-xanthophyll concentrate		15.0	30.0
Soybean meal	250.0	235.0	220.0
Wheat	385.2	385.2	385.2
Maize	200.0	200.0	200.0
Flax meal	30.0	30.0	30.0
Dried whey	30.0	30.0	30.0
Dried brewer’s yeast	60.0	60.0	60.0
Monocalcium phosphate	10.0	10.0	10.0
Calcium carbonate	20.0	20.0	20.0
Sodium chloride	3.4	3.4	3.4
Mineral-vitamin premix ^1^	10.0	10.0	10.0
L-lysine	1.0	1.0	1.0
DL-methionine	0.4	0.4	0.4
Nutrient content (% dry matter)			
Dry matter	90.3	90.1	90.4
Crude protein	25.5	25.6	25.9
Crude ash	6.5	6.6	6.7
Ether extract	4.2	4.4	4.6
Crude fibre	4.7	4.9	5.0
ADF ^2^	6.9	6.5	6.9
NDF ^3^	24.1	24.1	27.9
ADL ^4^	2.1	1.9	2.0
Gross energy (MJ/kg)	17.1	17.1	17.0

^1^ Premix (Cargill Poland Ltd., Kiszkowo, Poland), provided per kg of feed: Ca—3.07 g; Fe—75 mg; Mn—10 mg; Cu—8 mg; Zn—25 mg; I—0.15 mg; Se—0.40 mg; vitamin A—15,000 IU; vitamin D3—1000 IU; vitamin E—80 mg; vitamin K3—3 mg; vitamin B1—4 mg; vitamin B2—4 mg; vitamin B3—15 mg; vitamin B6—6 mg; vitamin B12—55 µg; biotin—200 µg; folic acid—1 mg; choline—750 mg; calcium D-pantothenate—10 mg; antioxidant—30 mg. ^2^ Acid detergent fibre. ^3^ Neutral detergent fibre. ^4^ Acid detergent lignin.

**Table 2:** Sequences of primers (5′-3′) used for qPCR amplification of mucin and β-actin genes.

Gene	Primer	Forward	Product Length (Base Pairs)	Reference
MUC2	Forward	CAGAGTGCATCAGTGGCTGT	242	[19]
	Reverse	CCCGTCGAAGGTGATGTAGT		
MUC3	Forward	AACTGCGACTGGGGCACCCAGAAA	366	[19]
	Reverse	AAAACCGTTTTTGTGTTAGTAT		
β-actin	Forward	AACTGGGACGATATGGAGAAGATTT	252	[19]
	Reverse	TGGGCACAGTGTGGG TGA		

**Table 3:** Growth performance and relative weight of internal organs of rats fed diets supplemented with 1.5% and 3% alfalfa PXC.

	PXC (%)			SEM	*p* Value	Contrasts	
	0	1.5	3			Linear	Quadratic
Feed intake (g)	526	527	547	6.10	0.308		
Body weight gain (g)	154	160	165	2.70	0.264		
Feed efficiency (g/g)	3.4	3.3	3.3	0.04	0.281		
Relative organ weight (g/100 g body weight)							
Heart	0.30	0.31	0.30	0.003	0.800		
Liver	5.03	4.91	4.78	0.045	0.078	0.022	0.078
Pancreas	0.41	0.41	0.41	0.013	0.991		
Spleen	0.24	0.25	0.25	0.005	0.763		
Kidneys	0.94	0.94	0.94	0.010	0.997		
Stomach	0.57	0.56	0.56	0.010	0.971		
Small intestine	3.12	3.13	3.02	0.039	0.486		
Caecum	0.52	0.47	0.47	0.013	0.198		

**Table 4:** Biochemical blood indices of rats fed diets supplemented with 1.5% and 3% alfalfa PXC.

	PXC (%)			SEM	*p* Value	Contrasts	
	0	1.5	3			Linear	Quadratic
Total protein (g/L)	66	65	64	0.8	0.552		
Albumin (g/L)	35	33	35	0.4	0.137		
Urea (mmol/L)	6.6	6.4	6.6	0.18	0.845		
Cholesterol (mmol/L)	1.0	1.5	1.8	0.18	0.129		
Triglycerides (mmol/L)	1.5	1.5	1.6	0.10	0.951		
HDL ^1^, mmol/L	0.8	0.8	0.8	0.03	0.574		
Glucose (mmol/L)	13.6	13.7	13.9	0.29	0.951		
Creatinine (µmol/L)	36.4	36.9	37.7	0.96	0.879		
Uric acid (µmol/L)	140 ^a^	139 ^a^	242 ^b^	19.7	0.040	0.031	0.040
Total bilirubin (µmol/L)	11.4 ^b^	5.3 ^a^	8.5 ^a,b^	1.01	0.039	0.245	0.039
AST ^2^, U/L	87	91	97	4.7	0.705		
ALT ^3^, U/L	55	49	52	1.4	0.289		
ALP ^4^, U/L	762	736	740	27.8	0.927		
ACP ^5^, U/L	33	31	33	1.0	0.806		
Cholinesterase (U/L)	313	294	332	9.7	0.289		
LDH ^6^, U/L	409	279	324	25.6	0.105		
CK ^7^, U/L	476	472	530	33.8	0.750		
Amylase (U/L)	635	623	631	15.3	0.951		
Chloride (mmol/L)	90	98	100	4.7	0.632		
Phosphorus (mmol/L)	3.3	3.2	3.1	0.06	0.373		
Iron (µmol/L)	43.7	39.6	46.8	1.40	0.104		
Calcium (mmol/L)	3.0	3.3	3.2	0.07	0.286		
Magnesium (mmol/L)	1.0	1.0	0.9	0.02	0.843		

^a,b^ Mean values in rows with different superscript letters differ significantly (*p* ≤ 0.05). ^1^ High-density lipoprotein. ^2^ Aspartate aminotransferase. ^3^ Alanine aminotransferase. ^4^ Alkaline phosphatase. ^5^ Acid phosphatase. ^6^ Lactate dehydrogenase. ^7^ Creatine kinase.

**Table 5:** Parameters of caecal fermentation in rats fed diets supplemented with 1.5% and 3% alfalfa PXC.

	PXC (%)			SEM	*p* Value	Contrasts	
	0	1.5	3			Linear	Quadratic
Caecal digesta (g/100 g body weight)	2.82	2.73	2.90	0.055	0.477		
Digesta pH	6.47	6.51	6.61	0.038	0.315		
β-glucuronidase (U/g)	51.9	86.6	77.9	12.98	0.534		
Short-chain fatty acids (µmol/g)							
Acetic	31.5	31.4	28.3	0.83	0.204		
Propionic	5.45	5.75	5.12	0.147	0.220		
Isobutyric	0.43	0.46	0.58	0.013	0.364		
Butyric	19.9	17.5	18.3	0.77	0.466		
Isovaleric	0.12	0.16	0.21	0.016	0.057	0.017	0.057
Valeric	0.43	0.44	0.45	0.011	0.829		
Total SCFA	57.8	55.7	55.8	1.11	0.188		
Amines (µmol/g)							
Methylamine	0.016	0.018	0.016	0.0009	0.481		
Putrescine	0.011	0.010	0.011	0.0005	0.725		
Cadaverine	0.009	0.010	0.011	0.0022	0.785		
Tyramine	0.017	0.018	0.018	0.0012	0.670		
Spermidine	0.009	0.010	0.009	0.0004	0.705		

**Table 6:** Morphological parameters of the caecum and colon of rats fed diets supplemented with 1.5% and 3% alfalfa PXC.

	PXC (%)			SEM	*p* Value	Contrasts	
	0	1.5	3			Linear	Quadratic
Caecum							
Crypts depth (µm)	197	209	190	5.7	0.413		
Myenteron thickness (µm)	288	289	275	11.5	0.873		
Colon							
Crypts depth (µm)	305	310	348	8.1	0.059	0.031	0.059
Myenteron thickness (µm)	197	205	232	13.0	0.568		
Mucus layer thickness (µg dye/cm^2^)	87 ^b^	59 ^a^	63 ^a^	3.8	0.001	0.007	0.001

^a,b^ Mean values in the rows with different superscript letters differ significantly (*p* ≤ 0.05).

## Data Availability

The data presented in this study are available on request from the corresponding author.

## References

[1] Zanin V. (1998). A New Nutritional Idea for Man: Lucerne Leaf Concentrate.

[2] Gaweł E., Grzelak M. (2012). The effect of a protein-xanthophyll concentrate from alfalfa (phytobiotic) on animal production—A current review. Ann. Anim. Sci..

[3] Myer R.O., Cheeke P.R. (1975). Utilization of alfalfa meal and alfalfa protein concentrate by rats. J. Anim. Sci..

[4] Saunders R.M., Connor M.A., Booth A.N., Bickoff E.M., Kohler G.O. (1973). Measurement of digestibility of alfalfa protein concentrates by in vivo and in vitro methods. J. Nutr..

[5] Myer R.O., Cheeke P.R., Kennick W.H. (1975). Utilization of alfalfa protein concentrate by swine. J. Anim. Sci..

[6] Ognik K., Patkowski L., Grela E.R. (2012). Effect of a protein-xanthophyll concentrate from alfalfa and of genotype and sex of lambs on their blood redox profile. Bull. Vet. Inst. Pulawy.

[7] Pietrzak E., Grela E.R. (2015). The effects of adding lucerne protein concentrate to diets on the reproductive traits and blood metabolic profiles of sows and piglets. J. Anim. Feed Sci..

[8] Kwiecień M., Winiarska-Mieczan A., Danek-Majewska A., Kwiatkowska K., Krusiński R. (2021). Effects of dietary alfalfa protein concentrate on lipid metabolism and antioxidative status and dietetic value of muscles in broilers. Poult. Sci..

[9] Grela E.R., Ognik K., Czech A., Matras J. (2014). Quality assessment of eggs from laying hens fed a mixture with lucerne protein concentrate. J. Anim. Feed Sci..

[10] Grela E.R., Knaga S., Winiarska-Mieczan A., Zięba G. (2020). Effects of dietary alfalfa protein concentrate supplementation on performance, egg quality, and fatty acid composition of raw, freeze-dried, and hard-boiled eggs from Polbar laying hens. Poult. Sci..

[11] Vyas S., Collin S.M., Bertin E., Davys G.J., Mathur B. (2010). Leaf concentrate as an alternative to iron and folic acid supplements for anaemic adolescent girls: A randomised controlled trial in India. Public Health Nutr..

[12] Hadidi M., Khaksar F.B., Pagan J., Ibarz A. (2020). Application of ultrasound-ultrafiltration-assisted alkaline isoelectric precipitation (UUAAIP) technique for producing alfalfa protein isolate for human consumption: Optimization, comparison, physicochemical, and functional properties. Food Res. Int..

[13] Nicholson J.K. (2012). Host-gut microbiota metabolic interactions. Science.

[14] Louis P., Scott K.P., Duncan S.H., Flint H.J. (2007). Understanding the effects of diet on bacterial metabolism in the large intestine. J. Appl. Microbiol..

[15] AOAC (2011). Official Methods of Analysis of AOAC International.

[16] Barszcz M., Taciak M., Skomiał J. (2011). A dose-response effects of tannic acid and protein on growth performance, caecal fermentation, colon morphology, and β-glucuronidase activity of rats. J. Anim. Feed Sci..

[17] Taciak M., Barszcz M., Tuśnio A., Bachanek I., Pastuszewska B., Skomiał J. (2015). The effects of type of protein and fibre fermented in vitro with different pig inocula on short-chain fatty acids and amines concentration. J. Anim. Feed Sci..

[18] Smirnov A., Sklan D., Uni Z. (2004). Mucin dynamics in the chick small intestine are altered by starvation. J. Nutr..

[19] Amit-Romach E., Uni Z., Cheled S., Berkovich Z., Reifen R. (2009). Bacterial population and innate immunity-related genes in rat gastrointestinal tract are altered by vitamin A-deficient diet. J. Nutr. Biochem..

[20] Livak K.J., Schmittgen T.D. (2001). Analysis of relative gene expression data using real-time quantitative PCR and the 2^−ΔΔCT^ method. Methods.

[21] Anugwa F.O.I., Varel V.H., Dickson J.S., Pond W.G., Krook L.P. (1989). Effects of dietary fiber and protein concentration on growth, feed efficiency, visceral organ weights and large intestine microbial populations of swine. J. Nutr..

[22] Pond W.G., Varel V.H., Dickson J.S., Haschek W.M. (1989). Comparative response of swine and rats to high-fiber or high-protein diets. J. Anim. Sci..

[23] Woodman D.D., Evans G.O. (1996). Assessment of hepatotoxicity. Animal Clinical Chemistry: A Primer for Toxicologists.

[24] Yuan J.-P., Peng J., Yin K., Wang J.-H. (2011). Potential health-promoting effects of astaxanthin: A high-value carotenoid mostly from microalgae. Mol. Nutr. Food Res..

[25] Ames B.N., Cathcart R., Schwiers E., Hochstein P. (1981). Uric acid provides an antioxidant defense in humans against oxidant- and radical-caused aging and cancer: A hypothesis. Proc. Natl. Acad. Sci. USA.

[26] Xie Z., Huang J., Xu X., Jin Z. (2008). Antioxidant activity of peptides isolated from alfalfa leaf protein hydrolysate. Food Chem..

[27] Jaswir I., Noviendri D., Hasrini R.F., Octavianti F. (2011). Carotenoids: Sources, medicinal properties and their application in food and nutraceutical industry. J. Med. Plants Res..

[28] Blachier F., Mariotti F., Huneau J.F., Tomé D. (2007). Effects of amino acid-derived luminal metabolites on the colonic epithelium and physiopathological consequences. Amino Acids.

[29] Wallace R.J. (2004). Antimicrobial properties of plant secondary metabolites. Proc. Nutr. Soc..

[30] Avato P., Bucci R., Tava A., Vitali C., Rosato A., Bialy Z., Jurzysta M. (2006). Antimicrobial activity of saponins from Medicago sp.: Structure-activity relationship. Phytother. Res..

[31] Giovannucci E. (2001). Insulin, insulin-like growth factors and colon cancer: A review of the evidence. J. Nutr..

[32] Sjögren K., Liu J.-L., Blad K., Skrtic S., Vidal O., Wallenius V., LeRoith D., Törnell J., Isaksson O.G.P., Jansson J.-O. (1999). Liver-derived insulin-like growth factor I (IGF-I) is the principal source of IGF-I in blood but is not required for postnatal body growth in mice. Proc. Natl. Acad. Sci. USA.

[33] Roberton A.M., Lee S.P., Lindop R., Stanley R.A., Thomse L., Tasman-Jones C. (1982). Biliary control of β-glucuronidase activity in the luminal contents of the rat ileum, cecum, and rectum. Cancer Res..

[34] Ridlon J.M., Kang D.-J., Hylemon P.B. (2006). Bile salt biotransformations by human intestinal bacteria. J. Lipid Res..

[35] Zaripheh S., Erdman J.W. (2002). Factors that influence the bioavailability of xanthophylls. J. Nutr..

[36] Wlodarska M., Willing B., Keeney K.M., Menendez A., Bergstrom K.S., Gill N., Russell S.L., Vallance B.A., Finlay B.B. (2011). Antibiotic treatment alters the colonic mucus layer and predisposes the host to exacerbated Citrobacter rodentium-induced colitis. Infect. Immun..

[37] Chromek M., Arvidsson I., Karpman D. (2012). The antimicrobial peptide cathelicidin protects mice from Escherichia coli O157:H7-mediated disease. PLoS ONE.

[38] Gibson G.R., Macfarlane G.T., Cummings J.H. (1993). Sulphate reducing bacteria and hydrogen metabolism in the human large intestine. Gut.

[39] Shimotoyodome A., Meguro S., Hase T., Tokimitsu I., Sakata T. (2000). Short chain fatty acids but not lactate or succinate stimulate mucus release in the rat colon. Comp. Biochem. Physiol. A Mol. Integr. Physiol..

[40] Hedemann M.S., Theil P.K., Bach Knudsen K.E. (2009). The thickness of the intestinal mucous layer in the colon of rats fed various sources of non-digestible carbohydrates is positively correlated with the pool of SCFA but negatively with the proportion of butyric acid in digesta. Br. J. Nutr..

